# A Label-Free Fluorescent Sensor Based on Si,N-Codoped Carbon Quantum Dots with Enhanced Sensitivity for the Determination of Cr(VI)

**DOI:** 10.3390/ma15051733

**Published:** 2022-02-25

**Authors:** Jinyu Zhang, Cai Jing, Binsong Wang

**Affiliations:** Key Laboratory of Functional Inorganic Material Chemistry, Department of Environmental Science, School of Chemistry and Materials Science, Ministry of Education of the People’s Republic of China, Heilongjiang University, Harbin 150080, China; zhangjinyu0810@163.com (J.Z.); jingcai1216@126.com (C.J.)

**Keywords:** Si,N-codoped carbon quantum dots, hexavalent chromium, fluorescence sensor, mechanism attributed to FRET

## Abstract

A signal shut-off probe of Si, N-codoped carbon quantum dots (Si, N-CQDs) was exploited to detect Cr(VI) by fluorescence quenching without the aid of any biomolecules or labeling materials. The sensing system prepared the precursor of diacetone acrylamide and the silane coupling agent 3-aminopropyltriethoxysilane (KH-550) by a simple hydrothermal method, and the quantum yield is as high as 75% Si, N-CQDs. The fluorescence stability and microstructure of the Si, N-CQDs were studied. The Si, N-CQDs has a high sensitivity for detecting Cr(VI) with the linear range of 0–200 μM and the detection limit of 0.995 μM. The quenching mechanism of Si, N-CQDs is attributed to FRET.

## 1. Introduction

In recent decades, heavy metal pollution in natural water has become more and more serious due to the rapid development of modern industry, among which hexavalent chromium (Cr(VI)) is the most toxic pollutant in the water system [1]. As a non-essential element, Cr(VI) has harmful effects on the human body, leading to genotoxic effects (lung cancer, chromosomal effects, DNA strand breaks, etc.,), hemotoxic effects (kidney damage and nerve effects), and carcinogenicity [2,3]. Therefore, establishing an efficient detection strategy for Cr(VI) in water [4]. At present, there are many analytical techniques for Cr(VI), such as colorimetry [5], atomic absorption spectrometry (AAS) [6], inductively coupled plasma mass spectrometry (ICP-MS) [7], inductively coupled plasma atomic emission spectroscopy [8] and spectrophotometry [9], these methods have the advantages of accurate results and high detection sensitivity, while they have the disadvantages of complex detection process, cumbersome operation, expensive equipment, and large influence by the environment factors [10]. More importantly, they require laboratory conditions during the testing process, so it is difficult to conduct quick and routine field tests [11]. Therefore, there is an urgent need for efficient and sensitive identification of Cr(VI), which is not restricted by the site and does not require sample pretreatment.

Carbon quantum dots (CQDs), as fluorescent chemical sensors based on fluorescence, have attracted extensive attention of many researchers [12,13]. The carbon quantum dots first discovered by Xu during the purification and separation process of carbon nanotubes have the characteristics of broadband optical absorption, bright luminescence, good chemical and optical stability, high water solubility, simple preparation, and low preparation toxicity and good biocompatibility [14,15]. CQDs nanoparticles’ size, shape, and composition are determined by their precursors and synthesis pathways [16]. CQDs synthesis methods can be divided into two ways: (a) physical synthesis methods, such as arc discharge [17], laser ablation [18], and plasma induction [19]; (b) chemical synthesis methods, such as hydrothermal [20], electrochemical synthesis [21], solvothermal [22], microwave-assisted [23], and carrier-assisted [24]. Because of the advantages of cheap equipment, simple operation, low energy consumption, high selectivity, and one-step production without multiple controls, the hydrothermal method based on aquatic systems is one of the most widely used methods [25]. CQDs have been widely used in the detection of Cr(VI). For example, Jasjot Kaur et al. used ascorbic and ethylene diamine as precursors, N-CQDs were prepared by a one-step hydrothermal method, and the fluorescence quenching between N-CQDs and Cr(VI) was used to detect Cr(VI) in water samples. The quenching mechanism is proved to be static quenching [26]. Harshita Laddha et al. took glutathione as N, C, and S source, PEG-400 as C source, H3PO4 as a P source, and N-S-P-codoped carbon dots synthesized by microwave induced pyrolysis for detecting trace Cr(VI) in textile industry wastewater by using the excellent fluorescence quenching effect of Cr(VI) [27].

Although CQDs have great potential in terms of luminescence behavior and fluorescence detection, their relatively low fluorescence quantum yield has attracted the scientific community’s attention [13]. CQDs doped with different heteroatoms is a method to solve the low quantum yield [28,29,30,31,32]. Because the atomic radii and orbitals of these heteroatoms are comparable to carbon’s, they form new surface states that can capture exciting electrons, thereby increasing the density and fluorescence quantum yield [33,34,35,36]. Silicon-doped CQDs introduce silane chains on the surface of CQDs through self-polymerization or copolymerization with silane. Due to the existence of a large number of silane chains on the surface, it can maintain good dispersion in water and most organic solvents [37], thereby greatly improving quantum yield (47–88%) and the performance of single particles in liquid state is maintained, resulting in the sharp quenching of fluorescence, such as agglomeration and self-absorption, does not occur [38].

Here, we demonstrate a simple green method to prepare Si,N-codoped CQDs by one-step hydrothermal method using diacetone acrylamide (source of C, N) and KH-550 (source of Si), with a quantum yield of 75%. The fluorescence quenching effect of Cr(VI) on Si,N-CQDs is linear in the range of 0–200 µM, and the detection limit is 0.995 µM, which can quantitatively detect Cr(VI) in water. The quenching mechanism is attributed to FRET. It provides a theoretical basis for applying green, fast, efficient, and real-time fluorescent probes.

## 2. Materials and Methods

### 2.1. Materials

Diacetone acrylamide (Shanghai Meryer Chemical Technology Co., Ltd., Shanghai, China) and KH-550 (Guangdong Lvwei New Material Technology Co., Ltd., Guangdong, China) were used as starting materials. Other reagents used include, MgSO_4_•7H_2_O, NaCl, CaCl_2_, Pb(NO_3_)_2_, K_2_Cr_2_O_7_, Cd(NO_3_)_2_•4H_2_O (Weifang Co-create Chemical Co., Ltd., Weifang, China), KCl, FeCl_3_•6H_2_O, MnSO_4_, FeCl_2_•4H_2_O (Tianjin Kemiou Chemical Reagent Co., Ltd., Tianjin, China), Hg(NO_3_)_2_, AgNO_3_, CuSO_4_•5H_2_O (Shanghai Aladdin Biochemical Technology Co., Ltd., Shanghai, China), Cr_2_(SO_4_)_3_•6H_2_O (Guangzhou Desheng Chemical Co., Ltd., Guangzhou, China), and deionized water.

### 2.2. Preparation of Si,N-CQD_s_

The synthesis process was to add 0.201 g diacetone acrylamide and 0.2 mL KH-550 into 30 mL deionized water. Stir the solution in the magnetic stirrer for 20 min and store it in an oven at 180 °C for 5 h to obtain a pale yellow solution. The prepared Si,N-CQDs emitted blue light under ultraviolet light. The resulting pale yellow liquid was dried to get dry light yellow powder, stored at 4 °C.

### 2.3. Characterization

Use a TU-1810 spectrophotometer to record UV-visible spectra (Beijing Peress Analytics Co., Ltd., Beijing, China). The PL spectra of Si,N-CQDs were recorded with Tristar -3020 (Shanghai Jingpu Technology Co., Ltd., Shanghai, China) spectrophotometer. The transmission electron microscope (TEM) observation was performed on the JEM-2100EX microscope (Japan electronics co., Ltd., Tokyo, Japan). XRD spectra were carried out by using a D/max-ⅢB diffractometer from Jin Gong Instrument Technology Co., Ltd., Nanjing, China, with the Cu Kα radiation source (wavelength = 0.1542 nm) setting to 40 kV and 40 mA.

The Fourier transform infrared (FTIR) spectra were measured on the Spectrum One FTIR spectrophotometer. The oxidation state was studied by X-ray photon spectroscopy (XPS, AXIS, SupraShanghai Juna Technology Co., Ltd., shanghai, China) with Mg Ka radiation (1252.6 eV at 75 W). Time-resolved photoluminescence (TR-PL) spectra were measured by a time-correlated single-photon counting (TCSPC) system coupled with a photomultiplier tube (H1461P-11, Hamamatsu, Tokyo, Japan). The sample was excited by a 50 picosecond-pulsed diode laser at 369 nm (APiL037X, NKT Photonics, Copenhagen, Denmark). The time resolution of the TCSPC system is approximately 1 nanosecond.

### 2.4. Fluorescence Quantum Yield (QY) of Si,N-CQDs

According to previous research [39], the fluorescence yield of Si,N-CQDs was determined by quinine sulfate (solvent is 0.1 mol/L H_2_SO_4_;Q_R_ = 0.54) was used as a reference substance and was calculated by referring to the Formula (1) below:

Q = Q_R_ × (I_S_/I_R_) × (A_R_/A_S_) × (η_S_^2^/η_R_^2^) (1)

where Q stands for quantum yield, A stands for absorbance, and I stands for fluorescence intensity, η represents refraction coefficient of solvent (η_S_^2^/η_R_^2^ = 1), and subscript R and S represent reference quinine sulfate and sample carbon quantum dots respectively.

### 2.5. Detection of Cr(VI)

The Cr(VI) stock solution was prepared with K_2_Cr_2_O_7_ for fluorescence detection of Cr(VI) ions. Cr(VI) was detected using the following method: first, 1 mL of different metal ions of concentration 500 µM/L, including Cu^2+^, Mg^2+^, K^+^, Na^+^, Mn^2+^, Cd^2+^, Hg^2+^, Al^3+^, Ag^+^, Fe^3+^, Fe^2+^, Ca^2+^, Cr^3+^, and Cr(VI) were added to 5 mL of Si,N-CQDs (control) solution of concentration 0.1 mg/mL. The photoluminescence spectra of the mixed solution were recorded, and the fluorescence quenching degree of Cr(VI) was the highest, which was obviously higher than that of other metal ions. All tests were performed at the same room temperature. Different concentrations of Cr(VI) (0–800 µM/L) were mixed under gentle shaking to assess the detection effect of Si,N-CQDs sensor on Cr(VI) in 5 mL of a reference substance. It was observed that the fluorescence intensity of Si,N-CQDs declined gradually with the rise of Cr(VI) concentration.

## 3. Results and Discussion

### 3.1. Optical Properties of Si,N-CQDs

**Figure 1:**
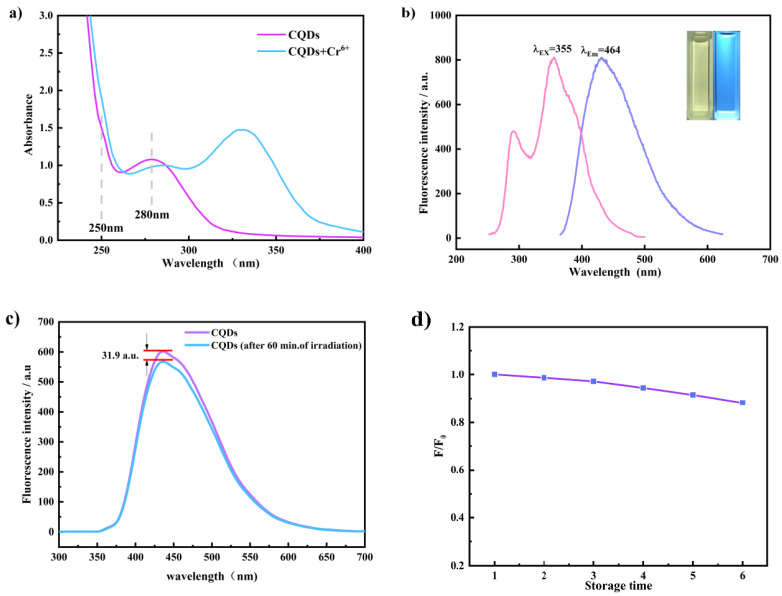
(**a**) The UV–Vis absorption spectrum of Si,N-CQDs in the absence and presence of Cr(VI). (**b**) The excitation and emission spectrum of Si,N-CQDs (insets show the corresponding colors under daylight and a 355 nm UV lamp). (**c**) The fluorescent spectrum of Si,N-CQDs before and after UV lamp irradiation at 355 nm for 60 min. (**d**) The effect of storage time on the fluorescence intensity of Si,N-CQDs.

Two absorption bands are observed in the UV-vis spectra of Si,N-CQDs aqueous solution (Figure 1a). The shoulder at 250 nm is caused by π-π* transitions in C=O, C=C, and C=N. Another maximum at 280 nm is owing to the typical n-π* transition in the carbonyl bond C=O [40].

As can be seen from Figure 1b, the maximum emission wavelength (λ_em_) is 464 nm at the excitation wavelength (λ_ex)_ of 355 nm, and the Si,N-CQDs produce blue fluorescence under 355 nm ultraviolet excitation. Using quinine sulfate as reference (λ_ex_ = 370 nm, QY = 54%), the average quantum yield (QY) of Si,N-CQDs was 75%.

In order to explore the optical stability which is one of the important optical properties of fluorescent probes, the fluorescence intensity of Si,N-CQDs was measured before and after irradiation with a 355 nm UV lamp for 1 h. Experimental results are shown in Figure 1c. Compared with unirradiated Si,N-CQDs, the irradiated Si,N-CQDs fluorescence intensity does not change significantly, which confirms that Si,N-CQDs has good optical stability [41].

In order to study the long-term stability of Si,N-CQDs, the fluorescence intensity of Si,N-CQDs placed in the dark was tested weekly. As is shown in Figure 1d, the fluorescence intensity of Si,N-CQDs decreases with the increase of storage time, and precipitation and suspended matter are not found in Si,N-CQDs solution. It is proved that the prepared Si,N-CQDs have good stability [42].

### 3.2. Structural Characterization of Si,N-CQDs

**Figure 2:**
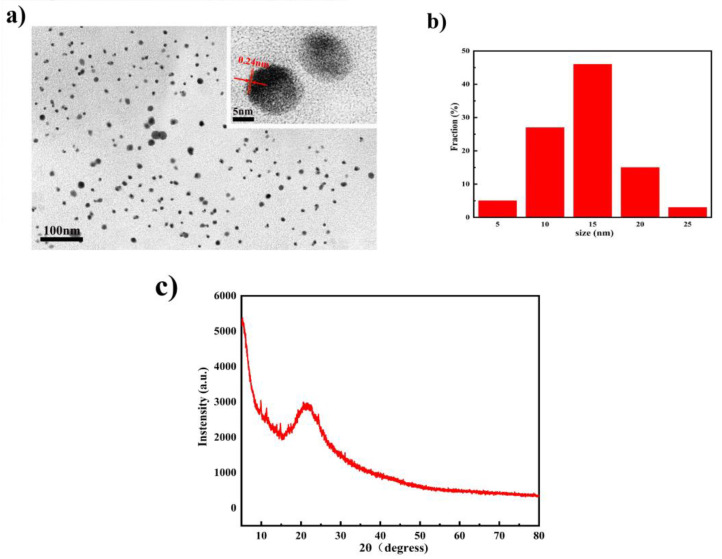
(**a**) The morphology of the Si,N-CQDs. (**b**) Size distribution histogram of Si,N-CQDs. (**c**) XRD diffractogram of Si,N-CQDs.

Use TEM to characterize the morphology of Si,N-CQDs, and the result is shown in Figure 2a. The prepared Si,N-CQDs are spherical and have good monodispersity in an aqueous solution. The size of Si,N-CQDs is small, with an average diameter of 14.8 nm. A lattice spacing of 0.24 nm was observed (inset in Figure 2a), which is approximately the graphite (100) plane, and the results revealed that Si,N-CQDs were prepared successfully [43,44]. The XRD patterns (Figure 2b) show the amorphous properties of Si,N-CQDs. The XRD pattern of Si,N-CQDs exhibited a broad peak at 2θ = 21.94°, which was attributed to the (002) plane of graphite [45].

**Figure 3:**
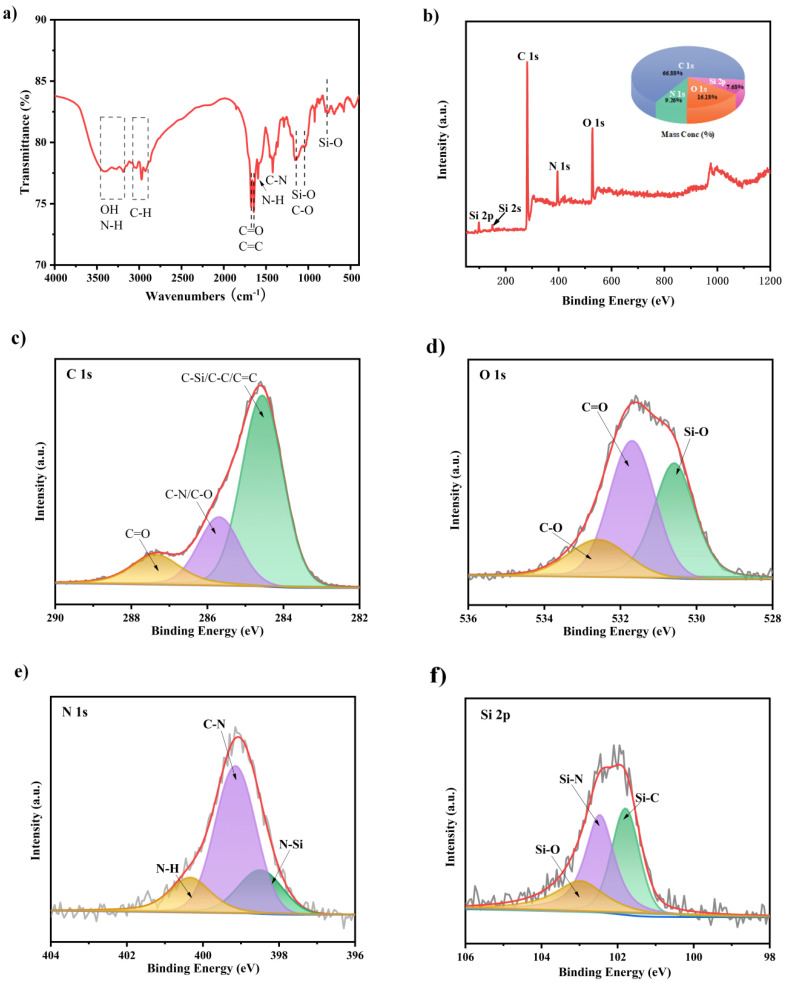
(**a**) FTIR spectrum of Si,N-CQDs. (**b**) XPS spectrum of Si,N-CQDs and high-resolution XPS C1s, O1s, N1s, and Si2p spectra (**c**–**f**).

The surface functional groups were characterized by FT-IR analysis. As is shown in Figure 3a, the surface functional groups of Si,N-CQDs were characterized by FT-IR. The broad bands at 3200 and 3448 cm^−1^ are due to O-H and N-H stretching vibrations. Peaks around 2972 cm^−1^ may be due to the stretching vibrations of C-H group. The peaks at 1678 and 1638 cm^−1^ are caused by stretching vibration of C=C and C=O, respectively. The small peak near 1592 cm^−1^ is caused by N-H bending vibration. The absorption at 1420 cm^−1^ is owing to the stretching vibration of C-N. The characteristic peak at 1148 cm^−1^ reflects the existence of C-O stretching vibration. The characteristic absorption bands of Si-O are shown at 1048 and 790 cm^−1^ [24,45,46].

The elemental composition and surface chemical states of Si,N-CQDs were further studied through XPS. Figure 3b shows the full scan of Si,N-CQDs. It can be seen that the four main peaks are located at 101.95, 284.60, 399.15, and 531.60 eV, representing C (1s), O (1s), Si (2p), and N (1s) respectively, indicating that N and Si atoms are successfully doped into Si,N-CQDs, and Si, C, N, and O accounted for 7.68% of Si, 66.88% of C, 9.26% of N, and 16.18% of O. As shown in Figure 3c, C (1s) spectrum has three peaks of 284.6, 285.7, and 287.4, which are C-Si /C-C(SP^3^)/C=C(SP^2^), C-N (SP^3^)/C-O(SP^3^), and C=O (SP^2^), respectively. Si, C, N, and O accounted for 7.68% of Si, 66.88% of C, 9.26% of N, and 16.18% of O. The spectra of N(1s) (Figure 3d) confirmed the presence of N in Si,N-CQDs, with different peaks corresponding to N-Si (398.5 eV), C-N (399.2 eV), and N-H (400.3 eV) respectively. The O1s (Figure 3e) peak is composed of three subpeaks at 530.6 eV, 531.7 eV, and 532.6 eV, respectively from Si-O, C=O, and C-O groups. As is shown in Figure 3f, the peaks at 101.8, 102.5, and 103.0 eV in Si (2p) spectrum support the existence of Si-C, Si-O, and Si-N groups, respectively [47,48].

### 3.3. Detection for Cr(VI)

**Figure 4:**
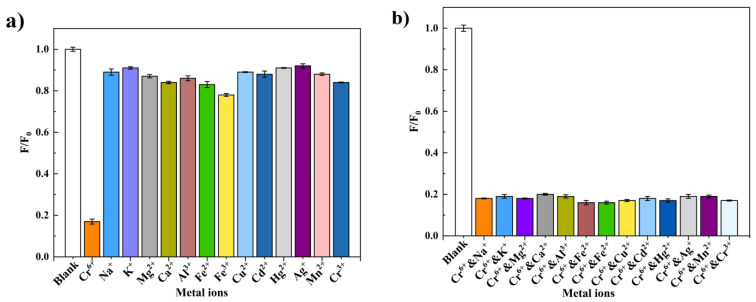
(**a**) The relative fluorescence intensity (F/F_0_) of Si,N-CQDs is added with various metal ions of 500 µM. (**b**) The relative fluorescence intensity (F/F_0_) of Si,N-CQDs-Cr(VI) system added with 500 µM various anions.

For the purpose of the study of the Si,N-CQDs to metal ions, the selectivity experiment of Si,N-CQDs was performed. The picture showed the fluorescence spectra of different metal ions in Si,N-CQDs solution and the influence of different metal ions on fluorescence intensity of Si,N-CQDs. In this paper, 14 common metal ions in water are selected. The concentrations were Cu^2+^, Mg^2+^, K^+^, Na^+^, Mn^2+^, Cd^2+^, Hg^2+^, Al^3+^, Ag^+^, Fe^3+^, Fe^2+^, Ca^2+^, Cr^3+^, and Cr(VI) at 500 µM, respectively. As is shown in the Figure 4a, the fluorescence quenching degree of Cr(VI) on Si,N-CQDs is the largest, and the fluorescence intensity of Si,N-CQDs is not reduced by the other 13 metals ions. It can be seen from the figure that in the presence of different metal ions, the fluorescence intensity of Si,N-CQDs is lower than F/F_0_ (F_0_ and F represent the fluorescence intensity of blank and Cr(VI) respectively). The figure proves that Si,N-CQDs have good specific selectivity for Cr(VI).

The influence of other metal ions on the stability of Si,N-CQDs-Cr(VI) system was investigated. Cu^2+^, Mg^2+^, K^+^, Na^+^, Mn^2+^, Cd^2+^, Hg^2+^, Al^3+^, Ag^+^, Fe^3+^, Fe^2+^, Ca^2+^, Cr^3+^, and Cr(VI) were respectively added to Si,N-CQDs-Cr(VI) system at a concentration of 500 µM. It can be seen from the Figure 4b that when other metal ions are added to Si,N-CQDs-Cr(VI) system. Cr(VI) still has the greatest degree of fluorescence quenching of Si,N-CQDs, and other metal ions have little effect on the fluorescence intensity of Si,N-CQDs-Cr(VI) system. The experimental results show that Si,N-CQDs as the fluorescent probe has good specific selectivity and anti-interference.

**Figure 5:**
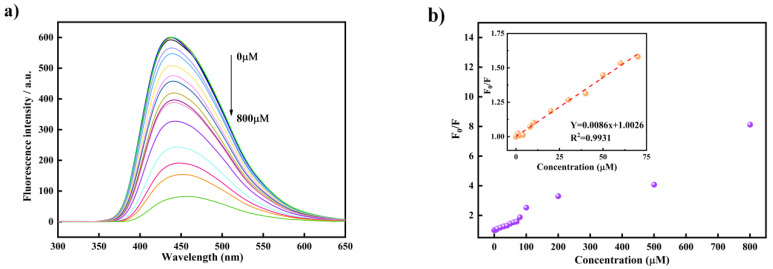
(**a**) The fluorescence emission spectrum of Si, N-CQDs is added with a series of Cr(VI) concentrations. (Illustration: Photograph of non-existent and present Si,N-CQDs of 800 µM Cr(VI) exposed to a 355 nm UV lamp). (**b**) The relative fluorescence intensity (F_0_/F) of Si,N-CQDs is added with different concentrations of Cr(VI). (Illustration: Linear relationship between F_0_/F and Cr(VI) concentration (0–200 µM)).

According to the selective fluorescence quenching of Cr(VI) by Si,N-CQDs, the response range of Si,N-CQDs to Cr(VI) was further discussed in this experiment. As shown in the Figure 5a, as the concentration of Cr(VI) increases, the fluorescence intensity of Si,N-CQDs gradually decreases. When the concentration reaches 500 µM, the fluorescence quenching degree of Si,N-CQDs solution reaches more than 80%. For the purpose of further determining the response range and detection limit of Si,N-CQDs, the fluorescence intensity data F_0_/F and Cr(VI) concentration are linearly fitted, and the results shown in Figure 5b. The fluorescence response concentration range of Si,N-CQDs solution to Cr(VI) is 0–200 µM, and the detection limit is 0.995 µM calculated according to formula 3/ơM of detection limit calculation. Among them, “ơ” refers to the standard deviation of fluorescence intensity measurement, and “M” refers to the slope of the linear equation, namely R^2^. There is a linear relationship between F/F_0_ and Cr(VI) concentration Y = 0.0086x + 1.00226 (R^2^ = 0.9982), indicating that Si,N-CQDs can be used as fluorescent probes to detect Cr(VI).

### 3.4. Quenching Mechanism

**Figure 6:**
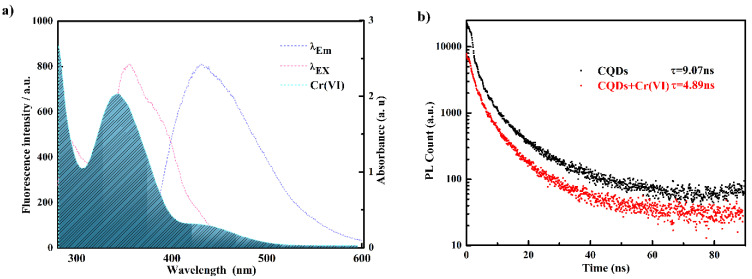
(**a**) The absorption spectrum of Cr(VI) and the excitation and emission spectra of Si,N-CQDs; (**b**) lifetime decays of Si,N-CQDs.

For the purpose of the study of the quenching mechanism of Si,N-CQDs, the following studies were carried out. As shown in Figure 6a, the UV absorption spectrum of Cr(VI) effectively overlaps the emission spectra and excitation of carbon dots, suggesting that the quenching mechanism is owing to the Forster resonance energy transfer (FRET) or inner filter effect (IFE) [49,50]. For the purpose of better understanding the quenching mechanism of Si,N-CQDs, the UV-vis absorption spectra of Si,N-CQDs with and without Cr(VI) were analyzed, as shown in Figure 1a. The addition of Cr(VI) results in red shift of the uV-vis absorption peak. This may be related to the interaction between Si,N-CQDs surface functional groups and Cr(VI). The results of the study show that the quenching mechanism of Si,N-CQDs is not IFE but a dynamic process [51]. For the purpose of further verifying the quenching mechanism of Si,N-CQDs by Cr(VI), in the absence or presence of Cr(VI), the fluorescence lifetime of Si,N-CQDs is measured at 355 nm, and the results of the study are shown in Figure 6. The fluorescence lifetime of Si,N-CQDs is 9.07 nm, and that of Si,N-CQDs+Cr(VI) is 4.89 ns [4,52]. The decrease in fluorescence lifetime confirms that the whole process is FRET.

## 4. Conclusions

In summary, Si,N-CQDs were successfully synthesized with acrylamide diacetone and KH-550 as raw materials. When exposed to a 355 anm UV lamp, it would emit bright blue fluorescence. The prepared carbon dots have a high quantum yield (QY = 75%) and good photostability. In addition, Si and N-CQDs showed good selectivity for Cr(VI), with the detection limit being 0.995 µM and the linear range being 0–200 µM (R^2^ = 0.9931), indicating that Si,N-CQDs have a good detection potential in fluorescence sensors.

## Data Availability

Not applicable.
